# Staff Attitude Towards Coercive Measures in Hospital and Community Psychiatric Settings

**DOI:** 10.3390/jcm14092886

**Published:** 2025-04-22

**Authors:** Rosaria Di Lorenzo, Francesca Mucchi, Nadia Magnani, Fabrizio Starace, Jessica Bonisoli, Carolina Bottone, Ilaria Ragazzini, Paola Ferri, Donatella Marrama

**Affiliations:** 1 Department of Mental Health and Drug Abuse, AUSL-Modena, 41121 Modena, Italy; d.marrama@ausl.mo.it; 2 Department of Biomedical, Metabolic and Neural Sciences, University of Modena and Reggio Emilia, 41125 Modena, Italy; mucchi.francesca@cert.ordine-opi.it (F.M.); paola.ferri@unimore.it (P.F.); 3 Adult Mental Health Functional Unit, ASL Toscana Sud-Est, 58100 Grosseto, Italy; nadia.magnani@uslsudest.toscana.it; 4 Department of Mental Health and Drug Abuse, ASL TO5, 10024 Moncalieri, Italy; starace.fabrizio@aslto5.piemonte.it; 5 School of Specialization in Psychiatry, Department of Biomedical, Metabolic and Neural Sciences, University of Modena and Reggio Emilia, 41125 Modena, Italy; j.bonisoli@ausl.mo.it (J.B.); c.bottone@ausl.mo.it (C.B.); ilaria.ragazzini@ausl.re.it (I.R.)

**Keywords:** coercion attitudes, healthcare professionals, psychiatric settings

## Abstract

**Background/Objectives**: The use of coercive measures in psychiatry is an ethically controversial issue. Staff attitude towards coercive measures could explain the different application frequencies of coercive measures across psychiatric services. **Methods**: We analyzed the attitude towards coercion held by professionals working in a psychiatric department using the Staff Attitude to Coercion Scale (SACS). We statistically evaluated the correlation between the SACS score and the demographic and work characteristics of professionals. **Results**: The most represented category of participants was nurses (73.03%). Most professionals worked in a Mental Health Community Service (MHCS) (72.09%). We reported a score of 41.9 ± 8.8 SD in total SACS and high scores in two SACS factors: “Coercion as offending” and “Coercion as care and security”. Professionals working in Service for Psychiatric Diagnosis and Care (SPDC) showed reduced scores in total SACS and the SACS dimension “Coercion as offending” score. Place of work, particularly “working in SPDC”, was statistically significantly associated with total SACS in a positive way and with the “Coercion as offending” score in a negative way in our regression multivariate test. **Conclusions**: Our professionals showed a predominantly critical and pragmatic attitude towards coercive measures. The professionals who are more frequently exposed to violent and aggressive behavior, such as those who work in SPDC, showed a reduced critical attitude towards coercion in comparison with those working in MHCS, suggesting that exposure to violence can shape the response of professionals.

## 1. Introduction

The use of coercive measures in psychiatry is an ethically controversial issue, long debated in mental health services. The term “coercion” refers to seclusion, restraint, and forced medication, but it can also include all involuntary treatments. A conflict exists between maintaining the patient’s autonomy and the safety of those in charge of patient care, fellow patients, and the patients themselves [1,2]. In some cases, coercive measures can represent the only way by which acute danger can be averted, with risk for the patient of severe consequences ranging from mental trauma and physical injuries to death [3,4].

In 2018, the University of Kent, in collaboration with Mental Health Europe (MHE), published a report entitled “Mapping and Understanding Exclusion: Institutional, Coercive and Community-Based Services and Practices Across Europe”, which updated a previous report on mental health legislation, involuntary admissions, and coercive measures application in 36 European countries, encompassing an investigation on the human rights of individuals who have been treated by mental health services, especially of those with psychosocial disabilities [5]. In Europe, the FOSTREN network (Fostering and Strengthening Approaches to Reducing Coercion in European Mental Health Services) includes researchers, experts, and clinicians, with the shared aim of addressing the issue of coercion in hospitals and communities across Europe [6]. The relevance of human rights in healthcare has been globally discussed, especially since the publication of the Convention on the Rights of Persons with Disabilities (CRPD) in 2008 [7]. The discussion on human rights has had a strong impact also on clinical practice in mental health services and challenges longstanding traditions of using coercive measures [8,9]. Furthermore, the WHO indicated that a legislative provision to eliminate coercion in mental health services and uphold the right to free and informed consent should be created [10]. Indeed, it is recognized that compulsory medical treatment, especially as part of coercion, can play a fundamental role in avoiding relapses and improving the progression of psychiatric illnesses, thus reducing the prevalence of institutionalization and improving the quality of life of the most vulnerable patients who are susceptible to social marginalization [11,12,13,14,15,16]. People with severe mental illness receiving compulsory community treatment were less likely to be victims of violent or non-violent crime, as suggested by another recent meta-analysis and meta-regression [17,18]. This study highlights the factors more frequently associated with involuntary treatments in psychiatry: the effects of human rights; recovery-oriented policies; environmental factors, including demographics; the availability of in-patient beds and clinical community-based resources; and peer or service culture [18]. However, it is crucial to recognize that this domain continues to be the subject of an ongoing discourse and a potential for improvement.

During the last few years, there has been considerable progress in the perception of coercion, no longer considered an indispensable tool but rather a measure of last resort, reserved for the most serious psychopathological conditions and the prevention of dangerous situations for both the patient and the professional [19]. A scoping review on health professionals’ attitudes towards coercive measures reveals a clear change in perspective over time [20], with an increased interest in staff attitudes towards coercion in mental healthcare, although there are different definitions concerning coercion. The implementation of ethics reflection groups, also called moral case deliberations, contributed to employees reporting a more critical attitude towards coercion, as highlighted by a Norwegian study [21].

Significant discrepancies between countries were observed in the legislation and policy for involuntary mental healthcare [22] and in the application of coercive measures, including seclusion rooms and mechanical restraints as well as forced administration of drug therapy, across different geographical regions [23,24,25,26]. A substantial body of research has highlighted that the utilization of coercive measures varies not only across different countries but also among different regions and hospitals within the same country, suggesting that the cultural and organizational context of each mental health service exerts a significant influence on coercive measure use in clinical settings as well as on the quality of treatments and therapeutic relationship between patients and staff members [27,28,29,30].

Socio-demographic variables of patients have been identified as relevant criteria for the application of coercive measures, but staff attitude towards coercion can also play a decisive role in coercive measure application, although this has not been fully investigated yet [31,32]. Moreover, different attitudes of staff towards coercive measures could explain the different frequency of coercive measure application across psychiatric services [33,34]. In fact, the subjective emotions and experiences of mental health professionals in confronting aggressive behavior could play a pivotal role in the patient relationship, potentially exacerbating or de-escalating aggressiveness [34,35,36,37,38,39,40].

A recent study conducted by Wullschleger et al. investigated the attitude towards coercion in 423 mental health professionals using the Staff Attitude to Coercion Scale (SACS), with a particular focus on the experience of violent events, showing that professionals’ insecurity feeling was significantly associated with less critical and more positive attitude towards coercion [39]. This study highlighted that attitudes towards coercive measures may be influenced not only by the experience of violence and feelings of insecurity concerning aggression but especially by the ability to safely manage potential danger from violent events [39]. The conclusion of this study, supported by the literature, indicates that healthcare professionals can evaluate the use of coercion as necessary only in potentially dangerous situations, although most of them expressed concerns regarding the privacy and autonomy of patients subjected to coercive measures [40]. Recently, many interventions have been studied to avoid coercion in psychiatry: organization, staff training, risk assessment, environment, psychotherapy, debriefings, and advance directives [1,2]. Among them, staff training and experience have been considered the conditions which can most influence the attitude towards coercion. Availability of trained and experienced staff and elimination of organizational barriers to a safe clinical environment should represent alternative non-coercive interventions for managing aggressive and violent behavior in the psychiatric clinical settings [41].

The PreVCo study (‘Prevention of Violence and Coercion’) investigated the effects of a structured program for the management of aggression and the prevention of violence and coercion in 55 psychiatric wards, suggesting that the main facilitators to prevent coercion can be represented by a receptive, collaborative ward culture, team spirit, and previous experiences in successful transformation processes, whereas barriers can include the demanding working situation, frequent fluctuation of staff and low team cohesion, obstacles in communication, a deficit-oriented perception of patients, and a low priority of the implementation process [42].

A few studies indicated that the inclusion in the staff of highly experienced mental health professionals can be an effective strategy for reducing the use of coercive interventions. This is supported by the evidence that work experience has a positive effect on improving attitudes towards reducing such interventions. Furthermore, enhancing staff members’ capacity to reflect on their own attitudes, emotions, and actions could result in a reduction in coercive interventions [43]. In accordance with an Indian study, the lack of resources could be one of the reasons for coercion, which could be counteracted by improving hospital resources and staff training in verbal de-escalation techniques [44], as a justification for coercion in public health crisis [45].

Nevertheless, up to now, staff attitudes towards coercion have not been fully assessed and little is known about staff attitudes towards emotions accompanying these measures [43]. There is limited knowledge of the agreement among mental healthcare professionals’ opinions on their use [30], and research results on staff attitudes and their role in the use of coercive measures are inconsistent.

Attitudes of mental health professionals towards the use of coercion are highly relevant concerning its use in mental healthcare, as mental health professionals have to deal with the ethical issues represented by dangerous situations for patients and others in which they can decide to use coercion or not within a legal framework. Therefore, the assessment of those attitudes and the professionals’ demographic and work setting characteristics is relevant for research in this field.

### Purposes of This Study

The purpose of this study is to analyze the attitudes towards coercion of professionals working in one psychiatric department using the Staff Attitude to Coercion Scale (SACS) and to evaluate the correlation between the SACS score and the demographic and work characteristics of the professionals to better understand the variables and covariates for the use of coercion and the factors involved in psychiatry.

## 2. Materials and Methods

### 2.1. Study Design, Period, and Setting

This observational, prospective, and single-center study is included in the national retrospective–perspective multicenter study promoted by the Italian Society of Psychiatric Epidemiology (SIEP), aimed at evaluating the staff attitudes towards coercive measures in different Italian regions (study on compulsory psychiatric treatments entitled “National multi-center study on factors related to the implementation of Compulsory Health Treatment in Mental Health Services”, Ethical Committee of Area Vasta Sud-Est Toscana, ID 22676, Prot. N° 59 of 19 July 2022).

Our sample was represented by all psychiatrists, nurses, nurse assistants, psychologists, and psychiatric rehabilitation technicians working in the Service for Psychiatric Diagnosis and Care (SPDC) ward in Baggiovara Civil Hospital (BCH) and in the Mental Health Community Services (MHCSs) in Modena, who agreed to complete the Italian version of the Staff Attitude to Coercion Scale (SACS), providing their informed consent. The SACS scale was submitted to all professionals who had been working for at least three months in the Department of Mental Health of Modena.

A total of 124 professionals work within the Department of Mental Health of Modena, distributed among different services as follows:

- In the SPDC: 6 psychiatrists, 27 nurses, 2 psychiatric rehabilitation technicians, 4 nursing assistants, and 1 psychologist.
- In the 3 MHCSs: 21 psychiatrists, 4 psychologists, 48 nurses, 1 nursing assistant, 2 educators, and 8 psychiatric rehabilitation technicians.

In the Department of Mental Health in Modena, only one SPDC is available for a catchment area of more than 700,000 people. The SPDC is the only public acute psychiatric ward, with 15 beds for involuntary and compulsory treatments. In accordance with the 180 and 833 Italian Laws, it is the only hospital ward where involuntary hospitalizations can be accepted and is in a general hospital (BCH). In a separate part of the SPDC ward of AUSL-Modena, two beds for 14–17 year-old minors are available. The SPDC is directly connected with the MHCSs for regular continuity of treatments between in- and out-patients. The study period was between June and December 2023. In 2023, we reported the following frequency of voluntary and involuntary hospitalizations in the SPDC: 222 voluntary hospitalizations (46%), 202 involuntary hospitalizations (42%), according to law 180/78, and 56 voluntary hospitalizations of minors (12%). The rate of involuntary hospitalizations per 10,000 inhabitants was 3.7%, which was higher than others applied in the Emilia Romagna region and in Italy [46]. In the SPDC, no mechanical restraints nor seclusion procedures were applied in accordance with local guidelines, but only forced medication was used in involuntary hospitalizations.

### 2.2. Staff Attitude to Coercion Scale (SACS)

SACS is an instrument used worldwide to explore the attitudes of different professionals towards coercion in mental health settings, developed in Norway in 2008 [47,48]. It is considered a useful and feasible tool [49], which has been validated in several languages, including Italian, German, Japanese, Polish, Portuguese, and Turkish [50,51,52,53,54,55,56]. SACS is a self-administered questionnaire comprising 15 items, with each item scoring from 1 to 5. The scoring system is as follows: 1 = strongly disagree; 2 = disagree; 3 = neither agree nor disagree; 4 = agree; 5 = strongly agree.

The items assessed by the SACS scale are the following:

1. Regressive patients need coercive treatment.
2. Coercive treatment is necessary from time to time to ensure safety conditions.
3. Coercive treatment can damage the therapeutic alliance.
4. Coercive treatment is a sign of failure of mental health services.
5. Coercive treatment can be compatible with empathetic and caring patient management.
6. Coercive treatment should be used more frequently.
7. Coercive treatment can prevent the development of dangerous situations.
8. Coercive treatment is traumatizing for patients.
9. Coercive treatment can be a safe option for patients with severe illness.
10. Patients who lack disease awareness need coercive treatment.
11. In patients who exhibit aggressive and dangerous behavior, the use of coercive treatment is necessary.
12. Coercive treatment is needed in dangerous situations.
13. Coercive treatment is used too often.
14. The use of coercive treatment increases under conditions of scarcity of means and resources.
15. The use of coercive treatment could be reduced by allocating more time for dialogue with patients.

In accordance with the validation study [47], the SACS items can be grouped into three dimensions or subscales or factors:

1. “Coercion as offending” (critical attitude). This dimension consists of the items that are most critical of the use of coercion and focuses on a wish to reduce the use of coercion since it can be potentially harmful and offending towards patients and the relationship between caregiver and patient.
2. “Coercion as care and security” (pragmatic attitude). This dimension consists of items focused on the use of coercion for security reasons and the opinion that using coercion is perceived as giving care. This attitude can represent a pragmatic view of the use of coercion as necessary for safety and security reasons in some circumstances when it is necessary for taking care of people who suffer from psychological crises.
3. “Coercion as treatment” (positive attitude). This dimension includes the items that say that more coercion should be used in mental health care when patients present regressive behavior and lack insight.

The three subscales overlap the earlier studies’ ones [57,58] and can be interpreted as different parts of a continuous dimension from “positive” to “negative” attitude towards the use of coercion [58]. Cronbach’s alpha for the total Staff Attitudes to Coercion Scale using all 15 items was 0.78, and for the three subscales, it ranged between 0.69 and 0.73 [47]. The internal consistency of the three-factor model of the Italian version of SACS was assessed through Cronbach’s alpha and yielded acceptable indexes, ranging from 0.64 to 0.77 [50].

If a total score is required, the scores of the items grouped in factor 1 (“Coercion as offending attitudes”) are inverted [47].

SACS is the only questionnaire measuring staff attitudes to the use of coercive interventions in mental health services. Its widespread use indicates that the questionnaire is perceived as feasible and useful [49].

### 2.3. Selected Variables

For each professional who completed the SACS, we collected the following variables: age, gender, profession, place of work (SPDC or MHCS), years of employment, and years of employment in the same service (specifying SPDC and/or MHCS). The SACS scores were correlated with the demographic and working variables of the professionals who completed the SACS form.

### 2.4. Statistical Analysis

We applied skewness and kurtosis tests for normality. We evaluated the mean (m) and standard deviation (SD) for continuous data, applying Student’s test for comparing means in case of normal distribution or the Kruskal–Wallis test in case of a non-normal distribution; we extrapolated percentages and applied Pearson’s chi2 for categorical variables.

We applied four multiple linear regression tests (forward and backward stepwise model) between the total SACS score, the score of “Coercion as offending”, “Coercion as care and security”, and “Coercion as treatment” of the SACS scale as dependent variables and the demographic and work variables of participants as independent variables.

We applied the Cronbach alpha for the total and 3-factor SACS.

A *p* value of 0.05 or less was considered statistically significant. All data were analyzed using the STATA Version 12 software (StataCorp LCC, College Station, TX, USA).

### 2.5. Ethical Considerations

This research was conducted in accordance with the principles of Good Clinical Practice, Declaration of Helsinki (version of the 64th WMA General Assembly, Fortaleza, Brazil, October 2013) [59]. This study was approved by the Ethics Committee of the Area Vasta Emilia Nord (917/2022/OSS/AUSLMO SIRER ID 5204—TSO Study) and was authorized by the AUSL of Modena (decision No. 1095 of 10/5/2023). After collecting data from our sample, they were anonymously reported in a database and successively statistically analyzed. Each professional who agreed to participate in this study and provided informed consent anonymously completed the SACS scale and the form with demographic and work variables, not reporting his/her name and surname but only a numeric code. Only the researchers involved in this study had access to the data.

## 3. Results

The participation in this study was high: 89 professionals out of 124 total (72%) agreed to participate: 68 nurses out of 75 (91%), 7 psychiatrists out of 27 (26%), 2 psychologists out of 5 (40%), 9 TRPs out of 10 (90%), 2 OSS out of 5 (40%), and 1 professional educator out of 2 (50%). In Table 1, the demographic and working data of the participants who completed the SACS scale are reported.

The most represented category of our professionals was nurses (*n* = 65; 73.03%), followed by psychiatric rehabilitation technicians (*n* = 9; 10.46%), psychiatrists (*n* = 7; 8.14%), psychologists (*n* = 2; 2.33%), nurse assistants (*n* = 2; 2.33%), and, finally, educators (1.16%). The average age of the professionals was 47.52 ± 10.47 SD. Females were more frequent (68.60%) than males (31.40%).

Regarding the place of work, a significant proportion of the professionals worked in the MHCSs, at 72.09% (62 professionals), followed by 24 professionals (24.91%) who worked in the SPDC. The data collected showed an average number of years of work corresponding to 22.84 ± 10.80 SD, with an average length of stay (also expressed in years) in the same psychiatric service of 13.24 ± 11.46 SD.

In the normality test, our sample did not present a normal distribution regarding the demographic and work variables: age (*p* = 0.0067), sex (*p* = 0.0000), profession (*p* = 0.000), place of work (*p* = 0.0002), years of employment (*p* = 0.0013), and years of employment in the same service (*p* = 0.0092). In Table 2, we report the SACS total and three-factor scores in relation to the demographic and work variables of the participants. We highlighted that only the total SACS (chi2 = 6.9; *p* = 0.0083; Kruskal–Wallis test) and the factor “Coercion as offending” (chi2 = 9.9, *p* = 0.0017; Kruskal–Wallis test) statistically significantly differed between the two places of work, SPDC and MCHSs.

**Figure 1:**
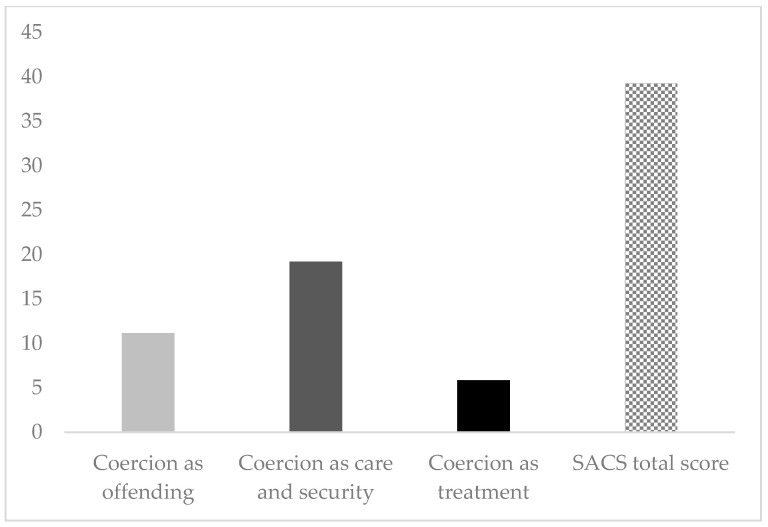
Total and 3-factor SACS scores.

As reported in Figure 1, in our sample, the most frequent dimension of SACS was represented by “Coercion as care and security”, which is characterized by a pragmatic attitude towards the practice, with a mean score of 19.2 ± 4.9 SD, followed by “Coercion as offending”, a critical attitude concerning coercive measures, with a mean score of 19.1 ± 3.7, and “Coercion as treatment”, or positive attitude concerning coercive measures, with the lowest mean score of 5.9 ± 2.3 SD. The total score of the SACS scale was 41.9 ± 8.8 SD, suggesting a predominantly critical and pragmatic attitude towards coercive measures.

As reported in Table 3, in the multiple linear regression analysis (forward and backward stepwise model), the variable “place of work in the SPDC ward” was significantly statistically associated with the dependent variable: negatively with the SACS factor ‘Coercion as offending’ (coeff.: −3.16; *p* = 0.001; IC 95% −4.95; −1.38) and positively with the total SACS score (coeff.: 6.94; *p* = 0.001; IC 95% 2.76; 11.12). Another work variable, “profession” was significantly statistically associated with the dependent variable: “nurse” (coeff.: −2.14; *p* = 0.019; IC 95% −3.91; −0.37) and “psychiatric rehabilitation technician” (coeff.: −3.39; *p* = 0.003; IC 95% −5.61; −1.16) were negatively associated with “Coercion as treatment”; “psychiatric rehabilitation technician” (coeff.: −9.43; *p* = 0.028; IC 95% −17.83; −1.04) was negatively associated with the total SACS score.

In our sample, Cronbach’s alpha for the total SACS was 0.8233; for factor 1, it was 0.6257; for factor 2, it was 0.7367; and for factor 3, it was 0.6113.

## 4. Discussion

The present study analyzed the attitude towards coercion of professionals working in one mental health service, composed of three community and one hospital settings, through the administration of the Staff Attitude Coercive Scale (SACS). SACS is one of the most used instruments for evaluating attitudes to coercion in workers and represents the only instrument now available that addresses these attitudes in general without focusing on a specific coercive measure [20]. This allows for comparing attitudes in different settings or countries that use different coercive measures [60,61]. SACS measures only explicit attitudes towards coercion and has been used in previous studies to examine associations between those attitudes and the incidence of coercive measures [49,54].

We obtained a good and acceptable value of Cronbach’s alpha for the total SACS score and a sufficient value of Cronbach’s alpha for the three dimensions of the SACS scale, which overlap with the results of validation studies [47,50], suggesting a good reliability of this scale translated into Italian.

Our sample was mainly represented by nurses, with a mean age of 47.52 years; most of them were females and had worked in MCHS for many years, in the majority of cases for more than 10 years in the same service, representing a sample with long work experience in psychiatric settings.

We reported the highest rate of participation in the study by nurses, while psychiatrists reported the lowest rate of adherence. This result is difficult to interpret, suggesting that psychiatrists either show partial interest in this or they are engaged in such demanding work that they do not have time to reflect on this dimension.

Our analysis highlighted that most of our participants did not consider coercive measures as a therapeutic instrument in psychiatric treatments, as shown by the lowest score of the “Coercion as treatment” factors reported by our sample. Our sample critically considered coercive measures as a violation of patient rights, although our professionals considered them a pragmatic approach necessary to ensure care in dangerous situations for both the patient and the staff. This finding overlaps with the literature. In fact, other authors reported a clear tendency to consider coercion as a violation of patients’ integrity with the risk of damaging therapeutic relationships, although they considered that coercion, in some cases, increased the safety of patients, staff, and others [62,63].

These results may be related to the long working experience (on average, 13 years) in mental health services of the participants, which can be correlated with a critical attitude towards coercion, according to some authors [62], who highlighted that staff coercion attitude can be secondary to limited experience in treating patients with aggressive and violent behavior.

The critical attitude towards coercive treatment can be interpreted as a concern for therapeutic alliance due to the risk of traumatizing the patient, removing him/her from mental health care. At the same time, coercive measures are interpreted as a signal of failure of mental health care or of the lack of therapeutic resources available. In contrast, a pragmatic approach towards coercive treatments can assume that coercion can represent a safe option for patients with severe psychiatric disorders, useful to prevent dangerous situations [47,48,49], which can explain the high rate of involuntary hospitalizations in our setting [46].

These results could have been positively conditioned by the department guidelines that have totally suspended the application of mechanical restraints in psychiatric hospitalizations after a profound cultural change lasting many years and supported by numerous training courses and professionals’ in-depth meetings. In fact, no mechanical restraints have been applied in the SPDC from 2021 to now (seclusion has never been applied in the ward, and a seclusion room is not available in the ward). In our sample, the SACS total and 3-factor scores were conditioned only by two demographic and working variables of our participants: “place of work” and “profession”, in particular, “nurse“ and “psychiatric rehabilitation technician”. In fact, the SACS total score and factor “Coercive as offending patient” statistically significantly differed between professionals working in the MHCSs and SPDC. Our study did not highlight any difference in attitude to coercive measures between sexes and among the different work periods, in contrast to other studies [49].

In accordance with our results, multiple linear regression tests indicated that “place of work” was the variable statistically significantly associated with the SACS score. In particular, the SACS total score was positively associated with SPDC place of work, whereas “Coercion as offending” was negatively associated with SPDC place of work. Our results suggest that professionals working in SPDC adopt a less critical, more pragmatic and positive attitude towards the utilization of coercive measures when compared to professionals who work in MHCS. This finding could be explained by the exposure to violence in an acute psychiatric ward such as an SPDC, where professionals are most frequently exposed to patient aggression and violence and, at the same time, need to maintain care in a safe work environment. Concurrently, these professionals exhibited heightened confidence in the management of situations that can represent a significant threat to the safety of both patients and professionals. This result overlaps with observations in the literature, which suggest that only healthcare professionals who do not frequently apply coercive instruments tend to adopt a more critical attitude towards their use [37,64]. In fact, as reported by another study, having less coercion experience predicted being less inclined to coercive attitude [30]. Our findings suggest that attitudes toward coercion in professionals are strongly influenced by workplace violence, which, with high emotional involvement, may shape the approach to the patient with aggressive behavior in a sort of unconscious vicarious and imitative learning, according to the definition by Bandura [65]. Nevertheless, this result indirectly represents that in acute psychiatric ward such as SPDC, violence can frequently occur and can represent a safety emergency.

Our results suggest that being a nurse or psychiatric rehabilitation technician is associated with a critical attitude towards coercion in comparison with other professions, similarly to other authors’ observations [30,40,41,43], indicating that an extensive relationship with patients, such as that required by these professions, can induce in the professionals an empathetic attitude with a reduced need for coercive measures.

Our sample predominantly comprised staff members in MHCS, where only a small percentage of patients exhibit such severe and acute altered conditions as to necessitate the implementation of coercive measures, whereas most of the patients treated need an empathic therapeutic relationship for maintaining long-term treatments. Therefore, it was more natural and easier for staff to consider critically the application of coercive measures.

As highlighted by a recent review [20], evidence supports a bidirectional relationship between attitudes toward coercion and the actual use of coercion in clinical practice. This means that attitudes towards coercion might influence if and how often coercive measures are used, but the use of coercion in clinical practice can also influence the attitudes of mental health professionals towards its use. This is relevant in terms of existing approaches to reduce coercion, as changes in the attitudes of mental health professionals are often viewed as essential in reducing the use of coercive measures or that negative attitudes are a barrier to reducing coercion [64,66].

To achieve this, mental health services need specific interventions addressing staff attitudes towards coercion. Considering the relevance of attitudes towards coercion for reducing coercive measures, studies that address attitudes need solid theoretical foundations [67]. Attitudes towards coercion might be defined in terms of moral and normative values or in terms of the cognitive, emotional, and behavioral components [68,69]. A recent scoping review has suggested updating the SACS scale, including items focused on emotional aspect of attitudes as well as developing different versions of the SACS scale for other relevant stakeholders [20] to make this scale more complete and more reliable in assessing coercion attitude.

### Limitations and Advantages of the Study

The present analysis was conducted as part of a multicenter study, which included several Italian centers. The study is yet to be completed, but it is anticipated that it will provide more comprehensive data on the associations between attitudes towards coercion and the selected demographic variables, as well as other demographic variables, such as gender and professional role.

The main limitations of the study are represented by the following:

- Small sample size.
- Data collected retrospectively do not allow us to make any causal inference.
- The mono-centric study design, which is limited to a single hospital setting, restricts the generalizability of the findings.

The advantages of this study are represented by having investigated the attitude towards coercive measures in real-world settings, comparing hospital and community staff of a psychiatric service.

## 5. Conclusions

From our analysis on the attitude of healthcare workers towards coercive measures, it emerges that coercion is no longer considered a completely necessary tool to use in the most serious psychopathological conditions and in the prevention of dangerous situations for both the patient and the worker. In fact, staff pragmatically consider coercion as a tool to ensure safety but are concerned about the possible repercussions on the patient.

In light of our results, we conclude that professionals working in an acute ward need to be emotionally supported for exposure to aggressive and violent behavior in order to avoid their reactive and potentially positive attitude towards coercion, implementing supervision and meetings to facilitate awareness of their feelings and emotions. As a practical implication, mental health professionals should, however, be encouraged to be aware of existing attitudes and to critically reflect on how these might influence their clinical practice. At the same time, the mental health service should be aware of the need to provide professionals with the most appropriate organization and the right tools to prevent and safely manage aggressive behavior in a psychiatric setting.

However, there is still much to be achieved to protect the safety of everyone, patients and professionals, promoting the safety of the work environment, and at the same time ensuring ethical and effective care, even in the most at-risk environments such as SPDC wards. Methods for preventing coercion should be addressed for all professionals working in clinical psychiatry, and the establishment of a shared, multi-professional, therapeutic attitude should always be an important goal within a team.

Nevertheless, avoiding coercion in psychiatric settings, particularly in acute psychiatric wards, would allow both patients and professionals to avoid negative consequences, especially a symmetrical escalation of violence.

By implementing changes in the future, the use of coercive measures can be further reduced, with increasing attention to the ethical aspects of care, improving the quality of services and training of health professionals. Future research should be conducted to discover the real impact of professionals’ emotional and cognitive attitudes towards coercion on the implementation of coercive measures in psychiatry.

## Figures and Tables

**Table 1:** Demographic and work variables of the professionals who completed SACS.

Variables	
Profession, *n* (%)	
Nurse	65 (75.6)
Psychiatric rehabilitation technician	9 (10.5)
Nurse assistant	2 (2.3)
Educator	1 (1.2)
Psychiatrist	7 (8.1)
Psychologist	2 (2.3)
Total	86
Age, m ± SD	47.5 ± 10.5
Sex, *n* (%)	
Male	27 (31.4)
Female	59 (68.6)
Place of work, *n* (%)	
MHCS	62 (72.1)
SPDC	24 (24.9)
Years of employment, m ± SD	22.8 ± 10.8
Years of employment in the same service, m ± SD	13.2 ± 11.5

**Table 2:** SACS total and factor scores correlated with demographic and work variables.

Variables	SACS Total Score (m ± SD)	Coercion as Offending (m ± SD)	Coercion as Care and Security (m ± SD)	Coercion as Treatment (m ± SD)
**Profession**				
Nurse	10.2 ± 8.1	19.0 ± 3.6	17.8 ± 4.4	7.5 ± 2.3
Psychiatric Rehabilitation Technician	5.9 ± 6.9	20 ± 4.3	15.8 ± 3.4	6.3 ± 1
Nurse Assistant	6 ± 1.4	17.5 ± 6.4	15.5 ± 9.2	8.5 ± 6.4
Educator	5	23	17	6
Psychiatrist	13.4 ±12.3	18.6 ± 4.4	20 ± 7.3	8.6 ± 2.9
Psychologist	9.5 ± 20.5	20.5 ± 2.1	17 ± 1.4	5.5 ± 0.7
Total	9.9 ± 8.5	19.1 ± 3.7	17.7 ± 4.6	7.4 ± 2.3
**Statistical test** **Probability**	chi2 = 5.78 *p* = 0.3279 Kruskal–Wallis test	chi2 = 2.73 *p* = 0.7422 Kruskal–Wallis test	chi2 = 3.79 *p* = 0.5798 Kruskal–Wallis test	chi2 = 9.1 *p* = 0.1044 Kruskal–Wallis test
**Age (</≥ median)**				
<51 years	11.02 ± 7.77	18.55 ± 3.66	18.36 ± 4.39	7.43 ± 1.93
≥51 years	9.477 ± 9.03	19.47 ± 3.81	17.39 ± 4.83	7.59 ± 2.65
**Statistical test** **Probability**	chi2 = 29.9 *p* = 0.5183 Kruskal–Wallis test	chi2 = 33.4 *p* = 0.3508 Kruskal–Wallis test	chi2 = 32.0 *p* = 0.4142 Kruskal–Wallis test	chi2 = 25.9 *p* = 0.7224 Kruskal–Wallis test
**Sex**				
Male	10.2 ± 8.3	19.2 ± 3.6	18 ± 4.8	7.5 ± 2.0
Female	9.7 ± 8.7	19.1 ± 3.8	17.5 ± 4.6	7.4 ± 2.5
**Statistical test** **Probability**	chi2 = 0.006 *p* = 0.9366 Kruskal–Wallis test	chi2 = 0.06 *p* = 0.8115 Kruskal–Wallis test	chi2 = 0.018 *p* = 0.8946 Kruskal–Wallis test	chi2 = 0.2 *p* = 0.6176 Kruskal–Wallis test
**Place of work**				
MHCS	8.3 ± 7.6	19.9 ± 3.4	17.1 ± 4.3	7.1 ± 2.2
SPDC	14.1 ± 9.5	16.9 ± 3.7	19.2 ± 5.3	8.2 ± 2.6
**Statistical test** **Probability**	chi2 = 6.9 *p* = 0.0083 Kruskal–Wallis test	chi2 = 9.9 *p* = 0.0017 Kruskal–Wallis test	chi2 = 3.2 *p* = 0.0722 Kruskal–Wallis test	chi2 = 2.4 *p* = 0.1237 Kruskal–Wallis test
**Years of employment (</≥ median)**				
<25 years	10.2 ± 9.4	18.9 ± 3.9	17.9 ± 4.9	7.3 ± 2.3
≥25 years	9.6 ± 7.7	19.4 ± 3.6	17.5 ± 4.3	7.5 ± 2.4
**Statistical test** **Probability**	chi2 = 35.3 *p* = 0.4547 Kruskal–Wallis test	chi2 = 27.1 *p* = 0.8281 Kruskal–Wallis test	chi2 = 38.0 *p* = 0. 3341 Kruskal–Wallis test	chi2 = 37.6 *p* = 0.3495 Kruskal–Wallis test
**Years of employment in the same service (</≥ median)**				
<10 years	9.9 ± 9.5	19.4 ± 3.7	18 ± 5.1	7.3 ± 2.49
≥10 years	9.7 ± 7.3	18.8 ± 3.8	17.3 ± 4.1	7.487 ± 2.18
**Statistical test** **Probability**	chi2 = 31.1 *p* = 0.6584 Kruskal–Wallis test	chi2 = 31.9 *p* = 0.3393 Kruskal–Wallis test	chi2 = 35.8 *p* = 0.4295 Kruskal–Wallis test	chi2 = 35.5 *p* = 0.4443 Kruskal–Wallis test

**Table 3:** Multiple linear regression between the (total and 3-factor) SACS scores.

Variables			
	Coeff.	Probability	95% Conf. Interval
**Coercion as offending**			
**Place of work:** SPDC	−3.16	0.001	−4.95; −1.38
**Profession:** Nurse	1.39	0.337	−1.47; 4.25
Psychologist	1.93	0.501	−3.75; 7.60
Nurse Assistant	2.09	0.486	−3.86; 8.04
Psychiatric Rehabilitation Technician	2.13	0.241	−1.46; 5.72
Educator	4.43	0.248	−3.14; 11.99
**Coercion as care and security**			
Place of work	2.01	0.085	−0.28; 4.29
Profession	−0.73	0.121	−1.67; 0.20
**Coercion as treatment**			
**Place of work:** SPDC	1.04	0.066	−0.07; 2.14
**Profession:** Nurse	−2.14	0.019	−3.91; −0.37
Psychologist	−2.71	0.128	−6.23; 0.80
Nurse Assistant	1.25	0.502	−4.94; 2.43
Psychiatric Rehabilitation Technician	−3.39	0.003	−5.61; −1.16
Educator	−4.71	0.049	9.40; −0.03
**SACS total score**			
**Place of work:** SPDC	6.94	0.001	2.76; 11.12
**Profession:** Nurse	−5.79	0.089	−12.48; 0.89
Psychologist	−8	0.234	−21.28; 5.27
Nurse Assistant	13.44	0.058	−27.36; 0.48
Psychiatric Rehabilitation Technician	−9.43	0.028	−17.83; −1.04
Educator	−10	0.264	−27.70; 7.70

## Data Availability

The original contributions presented in this study are included in the article; further inquiries can be directed to the corresponding author, but due to privacy and ethical restrictions, individual data are unavailable.

## Abbreviations

The following abbreviations are used in this manuscript:

SACS	Staff Attitude to Coercion Scale
SPDC	Service for Psychiatric Diagnosis and Care
MHCS	Mental Health Community Service
