# Correction: Di Lorenzo et al. Staff Attitude Towards Coercive Measures in Hospital and Community Psychiatric Settings. *J. Clin. Med.* 2025, *14*, 2886

**DOI:** 10.3390/jcm14207418

**Published:** 2025-10-21

**Authors:** Rosaria Di Lorenzo, Francesca Mucchi, Nadia Magnani, Fabrizio Starace, Jessica Bonisoli, Carolina Bottone, Ilaria Ragazzini, Paola Ferri, Donatella Marrama

**Affiliations:** 1 Department of Mental Health and Drug Abuse, AUSL-Modena, 41121 Modena, Italy; d.marrama@ausl.mo.it; 2 Department of Biomedical, Metabolic and Neural Sciences, University of Modena and Reggio Emilia, 41125 Modena, Italy; mucchi.francesca@cert.ordine-opi.it (F.M.); paola.ferri@unimore.it (P.F.); 3 Adult Mental Health Functional Unit, ASL Toscana Sud-Est, 58100 Grosseto, Italy; nadia.magnani@uslsudest.toscana.it; 4 Department of Mental Health and Drug Abuse, ASL TO5, 10024 Moncalieri, Italy; starace.fabrizio@aslto5.piemonte.it; 5 School of Specialization in Psychiatry, Department of Biomedical, Metabolic and Neural Sciences, University of Modena and Reggio Emilia, 41125 Modena, Italy; j.bonisoli@ausl.mo.it (J.B.); c.bottone@ausl.mo.it (C.B.); ilaria.ragazzini@ausl.re.it (I.R.)

## Text Correction

**There were three errors in the original publication [1].**

(1) A correction has been made to the Abstract, regarding the definition of SACS score increase and decrease, which were reversed (**the correction is in bold**).

**Abstract**
**Background/Objectives**: The use of coercive measures in psychiatry is an ethically controversial issue. Staff attitude towards coercive measures could explain the different application frequencies of coercive measures across psychiatric services. **Methods**: We analyzed the attitude towards coercion held by professionals working in a psychiatric department using the Staff Attitude to Coercion Scale (SACS). We statistically evaluated the correlation between the SACS score and the demographic and work characteristics of professionals. **Results**: The most represented category of participants was nurses (73.03%). Most professionals worked in a Mental Health Community Service (MHCS) (72.09%). We reported a score of 41.9 ± 8.8 SD in total SACS and high scores in two SACS factors: “Coercion as offending” and “Coercion as care and security”. Professionals working in Service for Psychiatric Diagnosis and Care (SPDC) showed **increased** scores in total SACS and **reduced** the SACS dimension “Coercion as offending” score. Place of work, particularly “working in SPDC”, was statistically significantly associated with total SACS in a positive way and with the “Coercion as offending” score in a negative way in our regression multivariate test. **Conclusions**: Our professionals showed a predominantly critical and pragmatic attitude towards coercive measures. The professionals who are more frequently exposed to violent and aggressive behavior, such as those who work in SPDC, showed a reduced critical attitude towards coercion in comparison with those working in MHCS, suggesting that exposure to violence can shape the response of professionals.

(2) In the original publication [1], the means and standard deviation of the SACS score were incorrectly reported in Table 2, as published. The corrected Table 2 is reported below (**corrections are in bold**).
(3) In the original publication [1], there was a mistake in Figure 1 as published. The corrected Figure 1 appears below.

The authors state that the scientific conclusions are unaffected. This correction was approved by the Academic Editor. The original publication has also been updated.

## Figures and Tables

**Figure 1:**
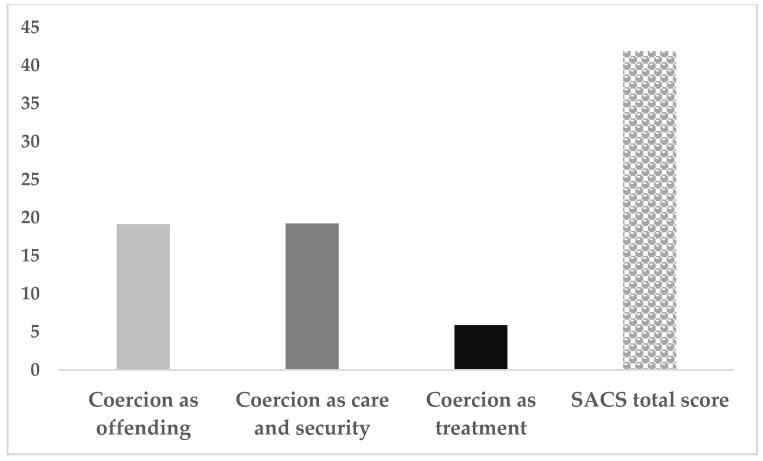
Total and 3-factor SACS scores.

**Table 2:** SACS total and factor scores correlated with demographic and work variables.

Variables	SACS Total Score (m ± SD)	Coercion as Offending (m ± SD)	Coercion as Care and Security (m ± SD)	Coercion as Treatment (m ± SD)
**Profession**				
Nurse	**42.2 ± 8.3**	19 ± 3.6	**19.4 ± 4.7**	**5.9 ± 2.2**
Psychiatric Rehabilitation Technician	**38.1 ± 7.2**	20 ± 4.3	**17.5 ± 3.3**	**4.5 ± 0.9**
Nurse Assistant	**38 ± 1.4**	**20.5 ± 2.1**	**17.5 ± 0.7**	**5 ± 1.4**
Educator	**36**	23	**20**	**3**
Psychiatrist	**46 ± 13.2**	18.6 ± 4.4	**20.8 ± 7.9**	**7.7 ± 2.7**
Psychologist	**39.5 ± 17.7**	**17.5 ± 6.4**	**16.5 ± 9.2**	**7.5 ± 6.4**
Total	**41.9 ± 8.8**	19.1 ± 3.7	**19.2 ± 4.8**	**5.9 ± 2.3**
**Statistical test** **Probability**	chi2 = 5.78 *p* = 0.3279 Kruskal–Wallis test	chi2 = 2.73 *p* = 0.7422 Kruskal–Wallis test	chi2 = 3.79 *p* = 0.5798 Kruskal–Wallis test	chi2 = 9.1 *p* = 0.1044 Kruskal–Wallis test
**Age (</≥ median)**				
<51 years	**43.02 ± 7.9**	**18.6 ± 3.6**	**19.8 ± 4.6**	**6.0 ± 2.0**
≥51 years	**41.4 ± 9.3**	**19.5 ± 3.8**	**19.1 ± 5.1**	**5.9 ± 2.5**
**Statistical test** **Probability**	chi2 = 29.9 *p* = 0.5183 Kruskal–Wallis test	chi2 = 33.4 *p* = 0.3508 Kruskal–Wallis test	chi2 = 32.0 *p* = 0.4142 Kruskal–Wallis test	chi2 = 25.9 *p* = 0.7224 Kruskal–Wallis test
**Sex**				
Male	**42.3 ± 8.4**	19.2 ± 3.6	**19.4 ± 5.0**	**6.1 ± 2.3**
Female	**41.7 ± 9.0**	19.1 ± 3.8	**19.1 ± 4.8**	**5.8 ± 2.3**
**Statistical test** **Probability**	chi2 = 0.006 *p* = 0.9366 Kruskal–Wallis test	chi2 = 0.06 *p* = 0.8115 Kruskal–Wallis test	chi2 = 0.018 *p* = 0.8946 Kruskal–Wallis test	chi2 = 0.2 *p* = 0.6176 Kruskal–Wallis test
**Place of work**				
MHCS	**40.3 ± 7.9**	19.9 ± 3.4	**18.6 ± 4.5**	**5.6 ± 2.1**
SPDC	**46.2 ± 9.5**	16.9 ± 3.7	**20.8 ± 5.5**	**6.6 ± 2.6**
**Statistical test** **Probability**	chi2 = 6.9 *p* = 0.0083 Kruskal–Wallis test	chi2 = 9.9 *p* = 0.0017 Kruskal–Wallis test	chi2 = 3.2 *p* = 0.0722 Kruskal–Wallis test	chi2 = 2.4 *p* = 0.1237 Kruskal–Wallis test
**Years of employment (</≥ median)**				
<25 years	**42.2 ± 9.6**	18.9 ± 3.9	**19.3 ± 5.4**	**5.9 ± 2.3**
≥25 years	**41.6 ± 8.1**	19.4 ± 3.6	**19.2 ± 4.4**	**5.8 ± 2.3**
**Statistical test** **Probability**	chi2 = 35.3 *p* = 0.4547 Kruskal–Wallis test	chi2 = 27.1 *p* = 0.8281 Kruskal–Wallis test	chi2 = 38.0 *p* = 0.3341 Kruskal–Wallis test	chi2 = 37.6 *p* = 0.3495 Kruskal–Wallis test
**Years of employment in the same service (</≥ median)**				
<10 years	**42.6 ± 10.4**	**19.3 ± 4.0**	**19 ± 4.4**	**6.4 ± 2.6**
≥10 years	**42.1 ± 7.7**	18.8 ± 3.8	**19.7 ± 5.5**	**5.8 ± 2.1**
**Statistical test** **Probability**	chi2 = 31.1 *p* = 0.6584 Kruskal–Wallis test	chi2 = 31.9 *p* = 0.3393 Kruskal–Wallis test	chi2 = 35.8 *p* = 0.4295 Kruskal–Wallis test	chi2 = 35.5 *p* = 0.4443 Kruskal–Wallis test
